# Supplementary material for: Sweat permeable and ultrahigh strength 3D PVDF piezoelectric nanoyarn fabric strain sensor
Source: Nat Commun. 2024 Apr 25;15:3509. doi: 10.1038/s41467-024-47810-7 (PMC11045766; doi:10.1038/s41467-024-47810-7)
Supplement: Supplementary file 1 — Supplementary information [file 41467_2024_47810_MOESM1_ESM.pdf]

Supplementary information

Sweat permeable and ultrahigh strength 3D PVDF piezoelectric nanoyarn fabric strain sensor

Wei Fan [1, *], Ruixin Lei [1], Hao Dou [1], Zheng Wu [1], Linlin Lu [1], Shujuan Wang [2], Xuqing Liu [3], Weichun Chen [1], Mashallah Rezakazemi [4, *], Tejraj M. Aminabhavi [5], Yi Li [6], Shengbo Ge [7, *]

[1] School of Textile Science and Engineering, Key Laboratory of Functional Textile Material and Product of Ministry of Education, Institute of Flexible electronics and Intelligent Textile, Xi'an Polytechnic University, Xi'an 710048, Shaanxi, China

[2] School of Chemistry, Xi'an Jiaotong University, Xi'an 710049, China

[3] State Key Laboratory of Solidification Processing, Center of Advanced Lubrication and Seal Materials, School of Materials Science and Engineering, Northwestern Polytechnical University, Xi'an, 710072, China

[4] Faculty of Chemical and Materials Engineering, Shahrood University of Technology, Shahrood, Iran

[5] Center for Energy and Environment, School of Advanced Sciences, KLE Technological University, Hubballi, 580 031, India and Korea University, Seoul 02841, Republic of Korea

[6] Department of Materials, University of Manchester Oxford Road, Manchester, M13 9PL, UK

[7] Co-Innovation Center of Efficient Processing and Utilization of Forest Resources, College of Materials Science and Engineering, Nanjing Forestry University, Nanjing 210037, China

Corresponding author Email: fanwei@xpu.edu.cn (W. Fan), geshengbo@njfu.edu.cn, mashalah.rezakazemi@gmail.com

Supplementary Information contains:

Supplementary Figures 1-10

Supplementary Tables 1-3

Supplementary References 1-6

Supplementary Movies 1−9

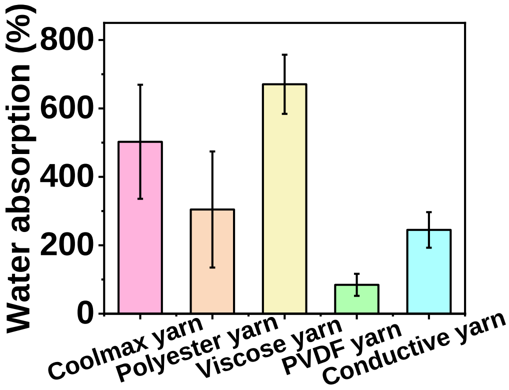

Supplementary Fig. 1. Comparison the water absorption of Coolmax yarn with polyester yarn, viscose yarn, conductive yarn and PVDF yarns.

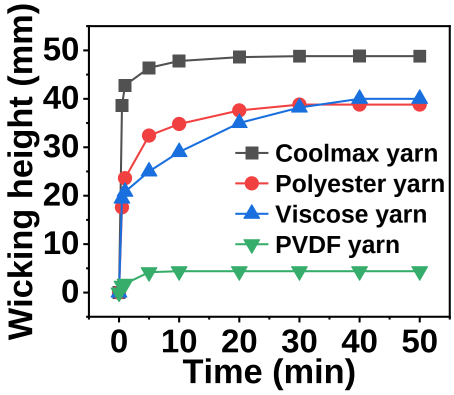

Supplementary Fig. 2. Comparison the wicking height of Coolmax yarn with polyester yarn, viscose yarn and PVDF yarns.

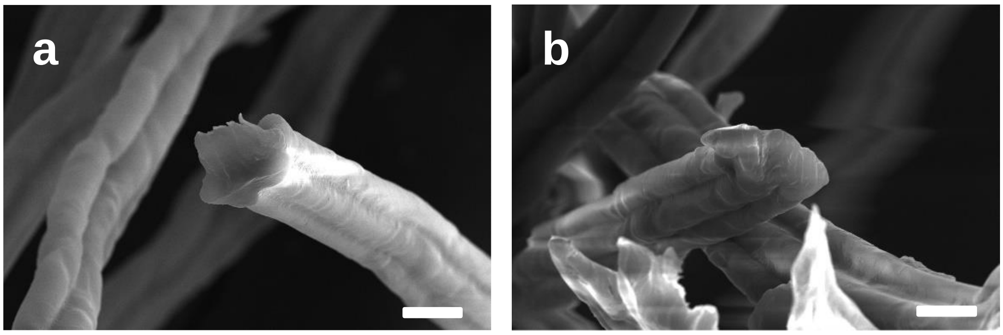

Supplementary Fig. 3. (a) Surface morphology and (b) cross-sectional morphology of the Coolmax yarn. The scale bar is 30 μm.

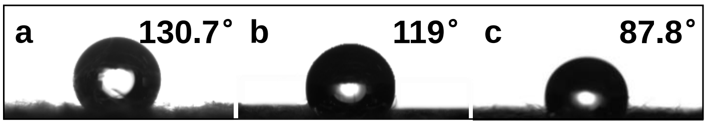

Supplementary Fig. 4. Water contact angle of (a) polyester yarn, (b) PVDF nanoyarn and (c) viscose yarn.

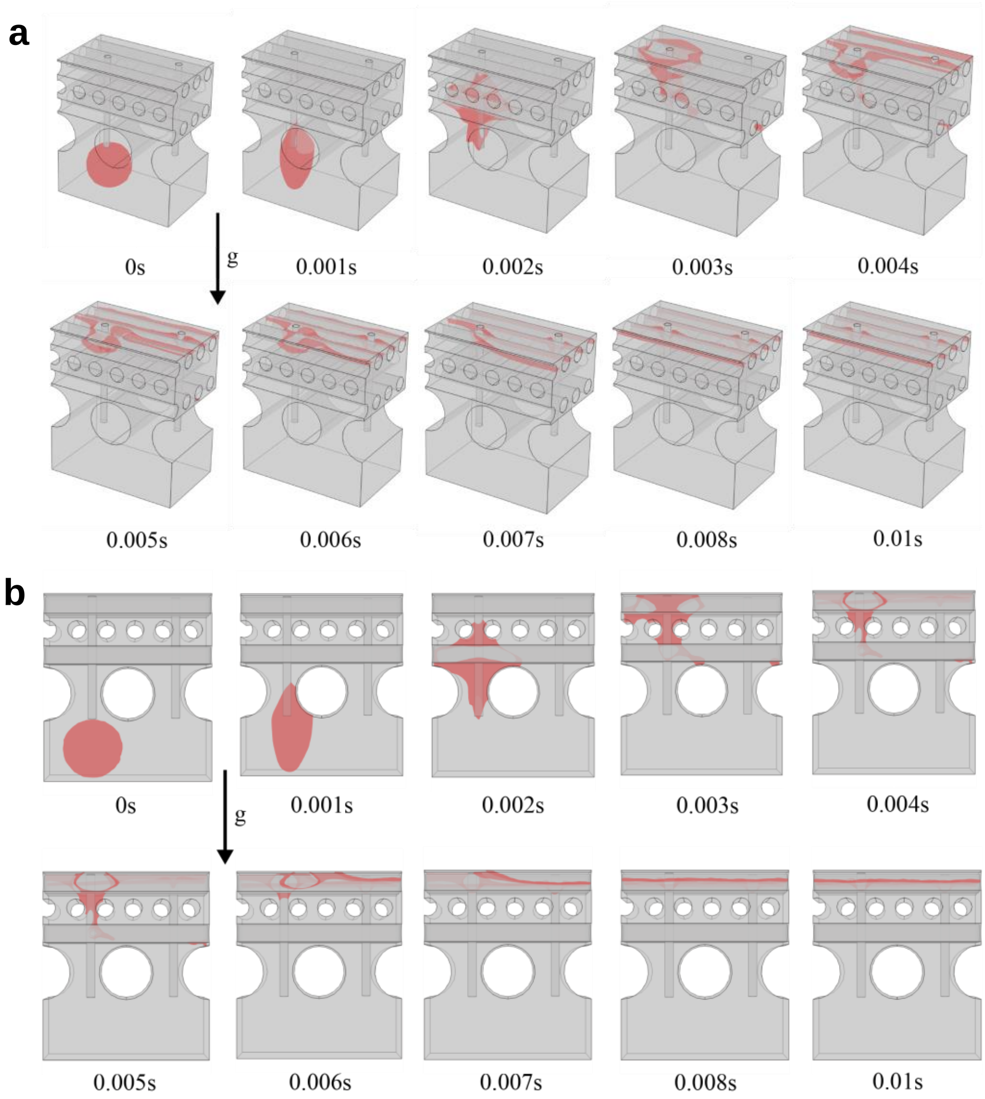

Supplementary Fig. 5. The anti-gravity unidirectional liquid transport phenomenon in the 3DPF in (a) isometric view and (b) main view angles.

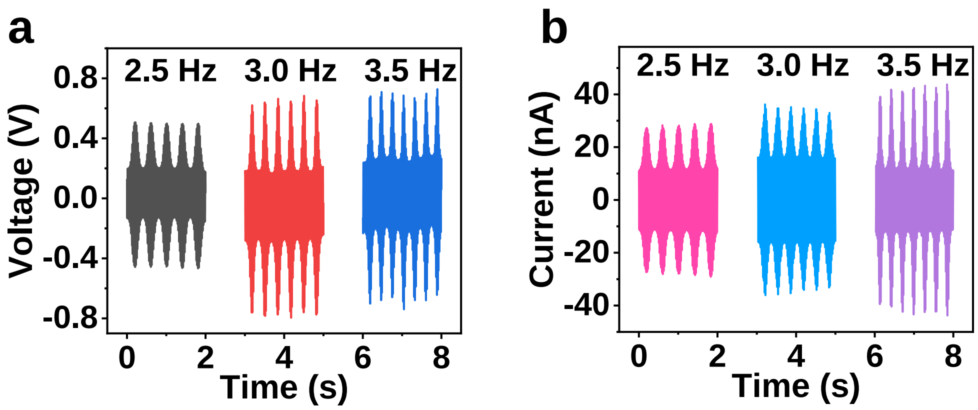

Supplementary Fig. 6. Output (a) voltage and (b) current of 3DPF at different frequencies.

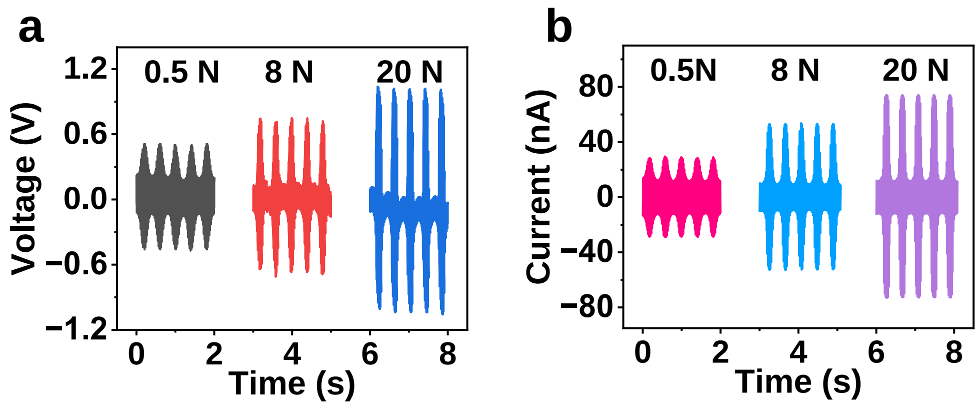

Supplementary Fig. 7. Output (a) voltage and (b) current of 3DPF after sweating.

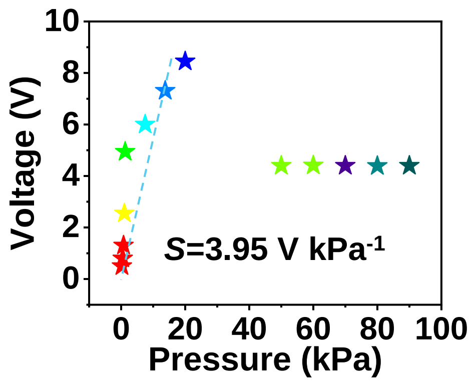

Supplementary Fig. 8. Voltage sensitivity of the 3DPF under sweating condition.

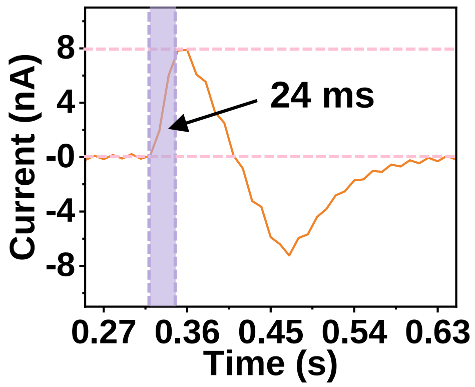

Supplementary Fig. 9. Response time of 3DPF after sweating (100 μL).

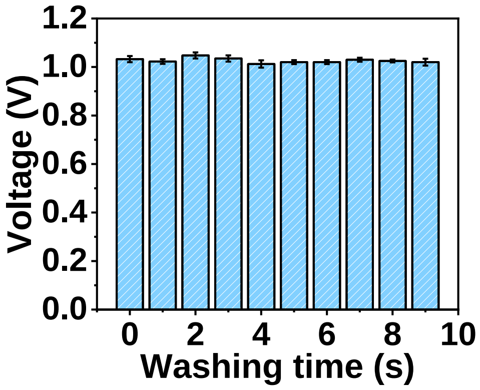

Supplementary Fig. 10. The electrical output of 3DPF after different washing times.

Supplementary Table 1. Comprehensive property comparison of different flexible piezoelectric sensors.

Sample	Structure	β-phase content (%)	Sensitivity	Tensile strength (MPa)	Ref.
PVDF	3Dorganic woven fabric	88.08	0.41 V kPa^{-1} 3.95 V kPa^{-1} (1.025 V N^{-1}– 9.875 V N^{-1})	46.0 ± 4.3	This work
PVDF/MOF	Nanofibrous membrane	75	0.118 V N^{-1}	< 25	[1]
(PVDF-$BaTiO_3$)/PA-11	Nanofiber membrane	69.2	107.52 mV N^{-1}	16.89±1.52 N	[2]
PVDF	Nanofiber webs	80.52	/	/	[3]
PVDF-ZnO	Nanofiber mats	87	/	/	[4]
PVDF/ZnO	Nanofiber mats	87	2.1795 mV N^{-1}	/	[5]
PVDF/ZnO	Nanofiber mats	81.4	2.37 mV kPa^{-1}	/	[6]

Supplementary Table 2. Technological parameters of the yarns.

Yarns (tex)						
Warp			Weft			Z-direction
Polyester yarn	HS yarn	Viscose yarn	Polyester yarn	Silvered nylon yarn	Viscose yarn	Coolmax yarn
30	20	236	30	19	236	39

Supplementary Table 3. Technological parameters of the 3DPF.

Fabric density (yarns cm^{-1})		Layers	Thickness (mm)	Grammage (g m^{-2})
Warp/Z-direction	Weft			
7	9	7	1.18	6

Supplementary References

1 Moghadam, B. H., Hasanzadeh, M. & Simchi, A. Self-Powered Wearable Piezoelectric Sensors Based on Polymer Nanofiber–Metal–Organic Framework Nanoparticle Composites for Arterial Pulse Monitoring. *ACS Appl. Nano Mater.* **3**, 8742-8752 (2020).

2 Kabir, H., Kamali Dehghan, H., Mashayekhan, S., Bagherzadeh, R. & Sorayani Bafqi, M. S. Hybrid fibrous (PVDF-BaTiO3)/ PA-11 piezoelectric patch as an energy harvester for pacemakers. *J. Ind. Text.* **51**, 4698S-4719S (2022).

3 Azmi, S., Hosseini Varkiani, S.-M., Latifi, M. & Bagherzadeh, R. Tuning energy harvesting devices with different layout angles to robust the mechanical-to-electrical energy conversion performance. *J. Ind. Text.* **51**, 9000S-9016S (2020).

4 Sorayani Bafqi, M. S., Bagherzadeh, R. & Latifi, M. Fabrication of composite PVDF-ZnO nanofiber mats by electrospinning for energy scavenging application with enhanced efficiency. *J. Polym. Res.* **22**, 130 (2015).

5 Sorayani Bafqi, M. S., Sadeghi, A.-H., Latifi, M. & Bagherzadeh, R. Design and fabrication of a piezoelectric out-put evaluation system for sensitivity measurements of fibrous sensors and actuators. *J. Ind. Text.* **50**, 1643-1659 (2019).

6 Mirjalali, S. et al. Multilayered Electrospun/Electrosprayed Polyvinylidene Fluoride+Zinc Oxide Nanofiber Mats with Enhanced Piezoelectricity. *Macromol. Mater. Eng.* **308**, 230009 (2023).
