# Supplementary material for: Sweat permeable and ultrahigh strength 3D PVDF piezoelectric nanoyarn fabric strain sensor
Source: Nat Commun. 2024 Apr 25;15:3509. doi: 10.1038/s41467-024-47810-7 (PMC11045766; doi:10.1038/s41467-024-47810-7)
Supplement: Supplementary file 3 — Description of Additional Supplementary Files [file 41467_2024_47810_MOESM3_ESM.pdf]

Description of Additional Supplementary Files

Supplementary Movie Legends:

Supplementary Movie 1. Weaving progress of 3DPF.

Supplementary Movie 2. The antigravity water transport experiment.

Supplementary Movie 3. COMSOL Multiphysics simulation of anti-gravity liquid transport experiment of 3DPF in isometric perspective.

Supplementary Movie 4. COMSOL Multiphysics simulation of anti-gravity liquid transport experiment of 3DPF in the main view.

Supplementary Movie 5. Simulating the piezoelectric testing process during sweating.

Supplementary Movie 6. Using piezoelectric fabric as trigger switch.

Supplementary Movie 7. Use wifi to transmit the alarm signal triggered by 3DPF.

Supplementary Movie 8. Use 4G to transmit the alarm signal triggered by 3DPF.

Supplementary Movie 9. Conjugated electrostatic spinning continues PVDF nanoyarns.
